# Host conservation through their parasites: molecular surveillance of vector-borne microorganisms in bats using ectoparasitic bat flies

**DOI:** 10.1051/parasite/2020069

**Published:** 2020-12-11

**Authors:** Tamara Szentiványi, Wanda Markotter, Muriel Dietrich, Laura Clément, Laurie Ançay, Loïc Brun, Eléonore Genzoni, Teresa Kearney, Ernest Seamark, Peter Estók, Philippe Christe, Olivier Glaizot

**Affiliations:** 1 Museum of Zoology 1014 Lausanne Switzerland; 2 Department of Ecology and Evolution, University of Lausanne 1015 Lausanne Switzerland; 3 Department of Medical Virology, University of Pretoria 0001 Pretoria South Africa; 4 AfricanBats NPC 0157 Pretoria South Africa; 5 UMR Processus Infectieux en Milieu Insulaire Tropical 97490 Sainte-Clotilde Reunion Island, France; 6 Ditsong National Museum of Natural History 0001 Pretoria South Africa; 7 Department of Zoology and Entomology, University of Pretoria 0083 Pretoria South Africa; 8 Department of Zoology, Eszterházy Károly University 3300 Eger Hungary

**Keywords:** *Bartonella*, blood-sampling, non-invasive method, Nycteribiidae, *Polychromophilus*, *Trypanosoma*

## Abstract

Most vertebrates host a wide variety of haematophagous parasites, which may play an important role in the transmission of vector-borne microorganisms to hosts. Surveillance is usually performed by collecting blood and/or tissue samples from vertebrate hosts. There are multiple methods to obtain samples, which can be stored for decades if properly kept. However, blood sampling is considered an invasive method and may possibly be harmful to the sampled individual. In this study, we investigated the use of ectoparasites as a tool to acquire molecular information about the presence and diversity of infectious microorganism in host populations. We tested the presence of three distinct vector-borne microorganisms in both bat blood and bat flies: *Bartonella* bacteria, malaria-like *Polychromophilus* sp. (Apicomplexa), and *Trypanosoma* sp. (Kinetoplastea). We detected the presence of these microorganisms both in bats and in their bat flies, with the exception of *Trypanosoma* sp. in South African bat flies. Additionally, we found *Bartonella* sp. in bat flies from one population in Spain, suggesting its presence in the host population even if not detected in bats. *Bartonella* and *Polychromophilus* infection showed the highest prevalence in both bat and bat fly populations. Single, co- and triple infections were also frequently present in both. We highlight the use of haematophagous ectoparasites to study the presence of infectious microorganism in host blood and its use as an alternative, less invasive sampling method.

## Abstract (fr)

La plupart des vertébrés hébergent une grande variété de parasites hématophages, qui peuvent jouer un rôle important dans la transmission de microorganismes à transmission vectorielle à leurs hôtes. La surveillance est généralement effectuée en prélevant des échantillons de sang et/ou de tissus sur des hôtes vertébrés. Il existe plusieurs méthodes pour obtenir des échantillons, qui peuvent être conservés pendant des décennies dans des bonnes conditions. Cependant, le prélèvement sanguin est considéré comme une méthode invasive et peut éventuellement être nocif pour l’individu prélevé. Dans cette étude, nous avons étudié l’utilisation d’ectoparasites comme outil pour acquérir des informations moléculaires sur la présence et la diversité des microorganismes infectieux dans les populations hôtes. Nous avons testé la présence de trois microorganismes distincts, transmis par des vecteurs, dans le sang et les mouches des chauves-souris : les bactéries *Bartonella*, *Polychromophilus* sp. (Apicomplexa) et *Trypanosoma* sp. (Kinetoplastea). Nous avons détecté la présence de ces microorganismes à la fois chez les chauves-souris et chez leurs mouches des chauves-souris, à l’exception de *Trypanosoma* sp. chez les chauves-souris sud-africaines. De plus, nous avons trouvé *Bartonella* sp. chez les mouches des chauves-souris d’une population en Espagne, ce qui suggère sa présence dans la population hôte même si elle n’est pas détectée chez les chauves-souris elles-mêmes. Les infections à *Bartonella* et *Polychromophilus* ont montré la prévalence la plus élevée dans les populations de chauves-souris et de mouches des chauves-souris. Des infections simples, doubles et triples étaient également fréquemment présentes dans les deux cas. Nous mettons en évidence l’utilisation d’ectoparasites hématophages pour étudier la présence de microorganismes infectieux dans le sang de l’hôte et son utilisation comme méthode alternative et moins invasive d’échantillonnage.

## Introduction

Bats are the second most diverse mammalian group and many of them have been recognised as keystone species, as they complete essential ecological functions, such as insect suppression, pollination and seed dispersal [43]. Besides their ecological roles, they are also important hosts of several diseases [10, 19, 34, 35, 52], and are the target of microorganism surveillance studies. Sampling of microorganisms can be done in many different ways. Strongly invasive (destructive) sampling includes collections of bat voucher specimens; invasive sampling involves blood sampling, hair sampling, wing punches, buccal or rectal swabbing; and non-invasive sampling includes the collection of faeces. This classification is, of course, somewhat arbitrary since invasiveness depends not only on the technique used but also on the handling time [42].

A recent study showed that 15% and 18% of bat species are Red Listed as threatened or data-deficient, respectively [26]. Russo et al. (2017) showed that the second main reason for voucher collection was disease studies (13%) after faunal surveys (65%). It was pointed out that voucher specimen collection, involving the euthanasia of thousands of bats within only two decades, might be problematic and unnecessary [72]. In their work, they proposed alternative techniques to avoid unnecessary killing of bats, including blood sampling. Nevertheless, collecting blood is operationally difficult and can be considered invasive. Furthermore, blood sampling often requires the use of chemical additives, such as sedatives, which may further increase health risks, during and after sampling, including higher risk of predation [15].

Several methods are used to collect blood samples from bats, including cardiac puncture using gauge needles or by venipuncture in the forearm or in the uropatagium to collect blood into capillary tubes [80]. It is recommended that the volume of the sample should not exceed more than 1% of the body weight of the sampled individual and cannot be taken more frequently than once a week [24].

The effects of blood sampling on bats are poorly known. Most of the research exploring the effects of blood sampling has been performed in birds and the results are controversial. It has been shown that blood sampling reduced bird survival by 21%–33% [8], although other studies found no such effect [1, 21, 39, 78]. Bird blood sampling can also induce behavioural changes, such as increased vocalisation [1]. Additionally, blood sampling is time-consuming, technically difficult, and handling individuals can significantly increase corticosterone levels [71, 98].

Only a few studies focused on the relationship between blood sampling and survival in mammals. In most cases, mammals did not show decreased survival after blood sampling, with some exceptions. For instance, Swann et al. (1997) did not find any significant decrease in survival for most of the tested small mammals. Only bled pocket mice (*Chaetodipus* sp.) had a lower survival when compared to un-bled specimens [85]. The only study focusing on bats used re-capture and PIT tag detection records and found no effect of blood sampling whether on short-term survival (after 14 days) or long-term survival (1-year return rate) in the big brown bats, *Eptesicus fuscus* [23]. Nevertheless, there is still a lack of knowledge about the effects of blood sampling on survival or behavioural changes in bats. Hence, additional efficient, non-invasive sampling procedures need to be explored where possible to minimise stress to the sampled individuals.

As an alternative to blood sampling, the use of haematophagous Heteroptera bugs has been suggested in ornithological research to non-invasively retrieve blood from individuals, e.g. for hormonal or for pathogen detection studies [2, 4, 5, 84]. It has also been successfully used in primates [90] and rabbits for hormonal studies [49, 92], as well as rabies serology in mice, under laboratory conditions [94]. A single study used haematophagous bugs to retrieve about 100 μL blood during a single feeding from captive bats in a doubly-labelled water experiment study for metabolic analyses [93]. We are not aware of any other studies using similar methods in the wild.

Bats harbour a high diversity of parasites and infectious microorganisms, including bacteria and viruses [10, 18, 31, 51, 73, 86, 87]. Here, we used molecular methods to reveal how effectively ectoparasites can be used for the detection of potentially infectious microorganisms, depending on their vectorial potential. For this, we sampled bat flies (Diptera: Nycteribiidae), one of the most common haematophagous ectoparasites of bats [18, 31]. Bat flies frequently feed on their hosts. For instance, some streblid species feed up to eight times an hour [27], increasing the probability of getting fresh host blood when collecting them. This was confirmed by the study of Witsenburg et al. (2015) who showed that host DNA was retrieved in 92.7% of bat flies [100]. However, the blood meal size of bat flies has never been estimated. Other ectoparasites of public health importance have been studied more extensively. For instance, the cat flea (*Ctenocephalides felis*) consumes on average 13.6 μL blood per day [20], whereas common bed bug (*Cimex lectularius*) males take on average 3.92 μL of blood per feeding [79].

We tested the presence of a vector-borne bacterium (*Bartonella* sp.), a malaria-like parasite (*Polychromophilus* sp.) and a trypanosomatid blood parasite (*Trypanosoma* sp.), both in bat flies and in their hosts’ blood. Bat flies are known to be vectors of *Polychromophilus* spp. [28], suspected vectors of *Bartonella* spp. [53, 74] but, to our knowledge, non-vectors of *Trypanosoma* spp. in bats.

We focused on the prevalence of these three vector-borne microorganisms in host blood and in ectoparasites in order to determine the reliability of using ectoparasites for their detection. Our aim was to explore a non-invasive technique that could replace blood sampling in blood microorganism surveillance studies, and to add new perspectives on using blood-sucking ectoparasites in other fields of bat research.

## Material and methods

Bat flies were collected from the Natal long-fingered bat (*Miniopterus natalensis*) in South Africa and from the common bent-wing bat (*M. schreibersii*) in Europe (Hungary, Italy and Spain), in 2015 and 2016 (Supplementary Table S1). In South Africa, permission was obtained to conduct research under Section 20 of the Animal Disease Act (Act No. 35 of 1984) from the Department of Agriculture, Land Reform and Rural Development of South Africa. This research was conducted with the approval of the University of Pretoria Animal Ethics committee (Project no. EC054-14 and EC059-14). Permits were obtained for bat sample collection from the South African provinces involved: the Department of Economic Development, Environment and Tourism Limpopo province directorate-wildlife permit nos. CPM006806, ZA/LP/83642 and ZA/LP/73972. Animal capture in Switzerland was conducted according to Swiss Animal Legislation (legislation number 2964).

Ectoparasite collection took place in the field, shortly after the capture of bats. Ectoparasites were found by blowing air into the fur and sweeping though the fur with forceps for about 2 min. Any parasites that were observed were removed from the hosts using forceps, which was in some cases dipped into ethanol. Bats were released after inspection. Parasites were preserved in 90% ethanol and afterwards morphologically identified in the lab using a stereomicroscope (Leica M205C in Switzerland and Nikon SMZ745T in South Africa) based on Theodor’s key [89] (Supplementary Table S1). Blood samples were collected from bats by venipuncture from wing vein or cardiac puncture based on standard sampling protocols [25].

DNA from host blood and ectoparasites was extracted using DNeasy Blood & Tissue Kits (Qiagen, Hilden, Germany), based on the protocol provided by the manufacturer. DNA samples were deposited in the Cantonal Museum of Zoology, Lausanne, Switzerland and the Department of Ecology and Evolution (DEE), University of Lausanne, Switzerland.

For *Bartonella* spp. detection, we targeted an approximately 800 bp fragment of the citrate synthase gene *gltA*, using 443F [7] and BhCS.1137n primers [57]. For *Polychromophilus* spp., a 704 bp fragment of the mitochondrial cytochrome b gene was amplified using the PLAS1 and PLAS2 primers for the first PCR round and the PLAS3 and PLAS4 primers for the second one [22]. For *Trypanosoma* spp., a fragment of 561 bp located in the small subunit (SSU) ribosomal RNA (rRNA) gene was amplified using the TRYF and TRYR primers for the first PCR round and the SSUF and SSUR primers for the second [58]. PCR protocols for each targeted microorganism are described in Supplementary File 1. Two PCRs were performed for each DNA sample. Positive PCR products were sent for Sanger sequencing to Microsynth, Switzerland. Sequences were analysed and edited in Mega7 [41]. Voucher sequences can be found in GenBank under the accession numbers: MT956920–MT956931 and MW007671–MW007713. Identification of ectoparasites and microorganism sequences was performed by nucleotide blast search in NCBI GenBank and a 92% of cut-off threshold was used for the identification of the sequences (Table S2).

We calculated the prevalence of infection from the bats and bat flies as the number of PCR-positive individuals over the total number of tested individuals. We then compared the estimates with the prevalence of infection calculated from bat flies using Chi-square tests for each targeted microorganism. To estimate prevalence of infection from bat fly data, we first considered all the tested flies (*n* = 101). Then, we combined results for flies collected on the same host (*n* = 57). For that, we considered the results positive (or negative) when all bat flies collected from the same host were positive (or negative). When both positive and negative bat flies co-occurred on the same host individual, we considered the result positive (consensus result). Statistical analyses and visualisation were performed using R 3.5.3 [65].

## Results

### Presence of infectious microorganisms in bats and bat flies

We tested the presence of three microorganisms (*Bartonella* spp.*, Polychromophilus* spp. and *Trypanosoma* spp.) in blood collected from the bats *Miniopterus schreibersii* (*n* = 35) and *M. natalensis* (*n* = 22; Supplementary Table S1). Additionally, we tested the presence of these potentially infectious microorganisms in their specific bat flies, *Nycteribia schmidlii* (*n* = 71, collected on *M. schreibersii*), and *N. schmidlii scotti* (*n* = 30, collected on *M. natalensis*). The datasets supporting the results can be found in Supplementary File 1 and in Table S1.

We collected an average of 1.4 ± 0.7 bat flies from *M. natalensis*, and 2.02 ± 1.4 bat flies from *M. schreibersii*. All tested bat individuals were infected with at least one bat fly (i.e. a prevalence of 100%).

The three microorganisms were found in both bat species, as well as in their specific bat flies, with the exception of *Trypanosoma* sp., which was not found in *N. schmidlii scotti* in South Africa. *Bartonella* infection was not detected in bats sampled in Spain, but this bacterium was found in their bat flies, suggesting *Bartonella* presence in the host population as well (Table 1).

**Table 1:** Prevalence (%) of *Bartonella* spp., *Polychromophilus* spp. and *Trypanosoma* spp. in bats and their bat flies.

Microorganisms	Bat host tested/infected	Prevalence (%)	Bat fly tested/infected	Prevalence (%)
	South Africa (MNAT)		(NSCO)	
*Bartonella*	22/11	50.0	30/17	56.7
*Polychromophilus*	22/9	40.9	30/6	20.0
*Trypanosoma*	22/2	9.1	30/0	0.0
	Hungary (MSCH)		(NSCH)	
*Bartonella*	9/3	33.3	17/9	52.9
*Polychromophilus*	9/6	66.7	17/6	35.3
*Trypanosoma*	9/3	33.3	17/6	35.3
	Italy (MSCH)		(NSCH)	
*Bartonella*	16/4	25.0	43/5	11.6
*Polychromophilus*	16/14	87.5	43/10	23.3
*Trypanosoma*	16/7	43.8	43/5	11.6
	Spain (MSCH)		(NSCH)	
*Bartonella*	10/0	0	11/2	18.2
*Polychromophilus*	10/6	60.0	11/2	18.2
*Trypanosoma*	10/3	30.0	11/9	81.8
	Total (Bats)		Total (Bat flies)	
*Bartonella*	57/18	31.6	101/33	32.7
*Polychromophilus*	57/35	61.4	101/31	30.7
*Trypanosoma*	57/15	26.3	101/13	12.9

Abbreviations: MNAT – *Miniopterus natalensis*; NSCO – *Nycteribia schmidlii scotti*; MSCH – *Miniopterus schreibersii*; NSCH – *Nycteribia schmidlii*.

### Co-infections and triple infections in bats and bat flies

**Figure 1:**
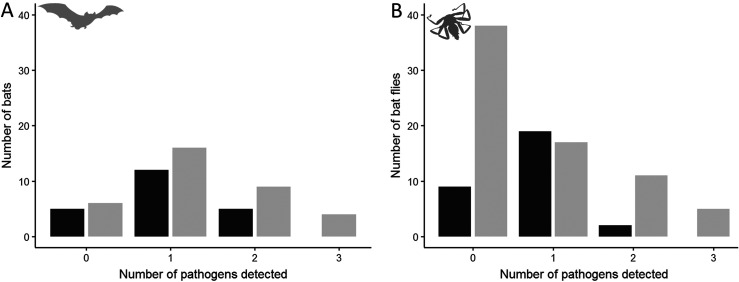
Number of detected vector-borne microorganisms in bats (A) and bat flies (B). Black colour corresponds to *Miniopterus natalensis* (A), and *Nycteribia schmidlii scotti* (B), whereas grey shows *Miniopterus schreibersii* (A) and *Nycteribia schmidlii* (B).

A total of 46 of the 57 bats (80.7%) were infected with at least one of the targeted microorganisms. Additionally, we found that 49% of bats (*n* = 28) were infected with one of them, 25% had co-infections (*n* = 14), and 7% triple infection (*n* = 4); whereas 19% of bats were uninfected (*n* = 11; Fig. 1A).

A total of 54 of the 101 tested bat flies (53.5%) carried at least one microorganism. Furthermore, 36% of the bat flies (*n* = 36) carried one of them, 13% two (*n* = 13), and 5% three (*n* = 5; Fig. 1B); while 47% of individuals (*n* = 47) were uninfected.

**Figure 2:**
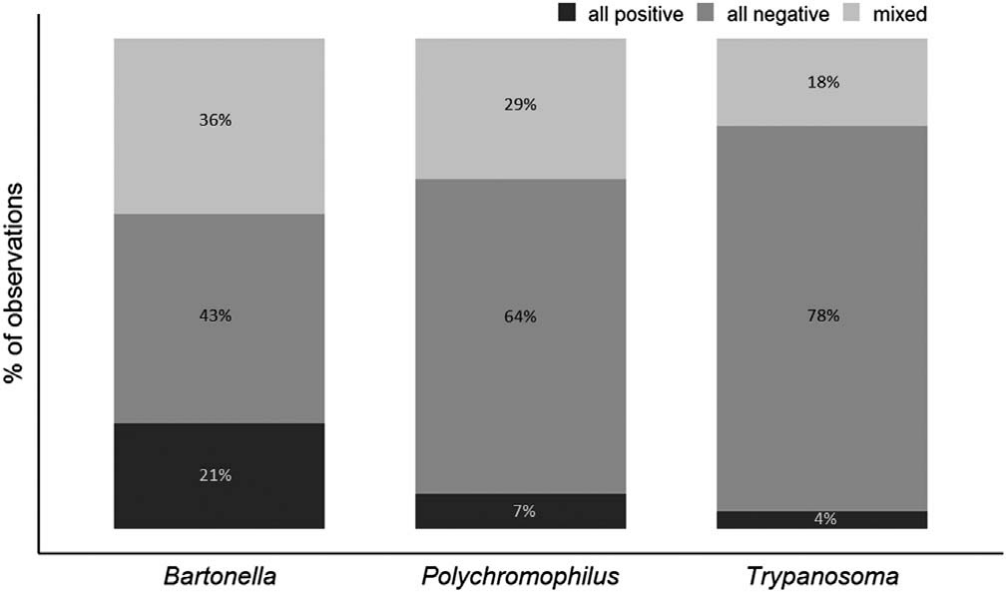
Prevalence of *Bartonella* spp., *Polychromophilus* spp., and *Trypanosoma* spp. infection in nycteribiid flies collected from 28 bats, which carried between 2 and 7 flies. Black: all flies are infected, dark grey: all flies are non-infected, light grey: both infected and non-infected flies occurred on the same host.

A total of 28 of the 57 infested bats (49%) hosted between 2 and 7 bat flies. PCR assays of these flies showed that both infected and non-infected flies co-occurring on the same host were found for 36%, 29% and 18% of these samples for *Bartonella* spp., *Polychromophilus* spp., and *Trypanosoma* spp., respectively (Fig. 2).

### Prevalence estimation

**Figure 3:**
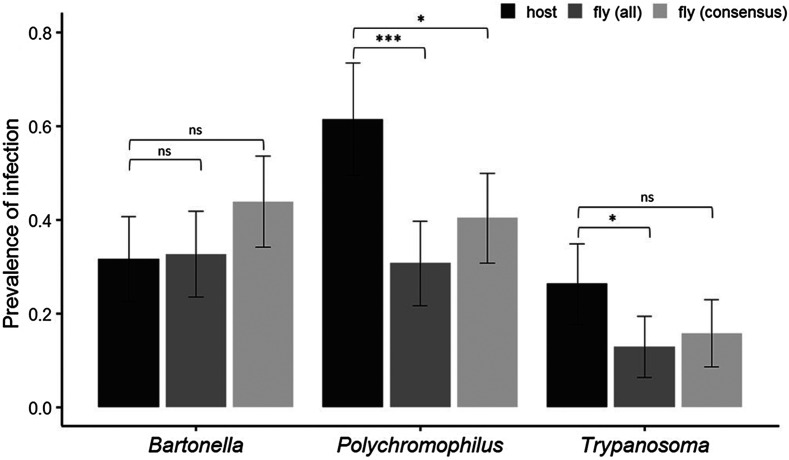
Comparison of detected microorganism prevalence (prevalence of infection) between bats and bat flies. Different bars represent hosts (black), all bat flies (dark grey), and consensus fly results, meaning that at least one infected fly individual was present on the host (light grey).

When all bat flies were considered (*n* = 101), prevalence estimated from bat hosts and their flies was significantly different for *Polychromophilus* (χ^2^_1_ = 14.128, *p* < 0.001) and for *Trypanosoma* (χ^2^_1_ = 4.517, *p* < 0.05). Indeed, prevalence estimated from hosts was 61% for *Polychromophilus* and 26% for *Trypanosoma*, however an almost 2-fold decrease was observed in the occurrence of both microorganisms in the corresponding bat flies (Fig. 3).

The estimated prevalence from hosts for *Bartonella* was 32% and no significant difference was seen when using data from all flies (33% ± 9%, χ^2^_1_ = 0.020, *p* = 0.888).

When a consensus on multiple bat fly results was done, the difference of prevalence estimates was reduced but still significant for *Polychromophilus* (χ^2^_1_ = 5.0542, *p* = 0.025, Fig. 3). In contrast, there was no significant difference for *Trypanosoma* (χ^2^_1_ = 1.9, *p* = 0.168), and still no difference for *Bartonella* (χ^2^_1_ = 1.8297, *p* = 0.176).

## Discussion

Our goal was to explore the possibility of using haematophagous ectoparasites for the detection of vector-borne microorganisms present in blood to replace invasive and possibly harmful blood sampling from hosts. Our study demonstrated the use of ectoparasites for detecting the presence of infectious microorganisms in their vertebrate hosts. However, our results are limited to the genus level; we found the presence of three infectious microorganisms, representing two parasites and one bacterium in bat flies over a broad geographical scale. This finding suggests that this technique could be expanded to other infectious microorganisms and/or hosts, including other mammals and non-mammalian organisms (e.g., birds or reptiles) infected by blood parasites and pathogens. In addition, applying this method to well-preserved museum collections could reveal historical pathogen dynamics and genetic patterns, as previously demonstrated for *Pseudogymnoascus destructans*, the causal agent of white-nose syndrome in bats [11, 101]. Additionally, museum collections are an important source of identifying other organisms in a wide range of taxa [6, 54, 91]. Historical collections might have degraded DNA, which could complicate pathogen and parasite detection; however, advances in new molecular techniques could overcome these problems in the future [14, 29, 60, 70, 88]. For further studies aiming at estimating infectious microorganism prevalence, it is important to consider testing multiple flies from the same host, as we observed variability of PCR-results among flies. The frequent co-occurrence of infected and non-infected flies on their hosts may indicate regular host switching or differences in infectious microorganism detectability in infected flies. These questions need further research in the future.

The detection of vector-borne microorganisms in bat flies was not significantly different when compared with prevalence from bat blood, for *Bartonella* (for both the total number of flies and consensus flies) nor for *Trypanosoma* (consensus flies). In contrast, ectoparasite sampling may fail to detect infectious microorganisms present in the host, as in the case with *Trypanosoma* sp. in *N. schmidlii scotti* in our study. *Trypanosoma* infection was present in the bats in South Africa but not in their bat flies *N. schmidlii scotti*. Our results also indicated that detection is independent of the vectorial capacity of the bat flies, as we detected the presence of *Trypanosoma* sp. in flies in three out of four sampled populations. Independent of the approach used, we found that the prevalence of *Polychromophilus* in flies was always lower than from bat hosts. This may be the result of lower detectability of this parasite in ectoparasites, which might be linked to either lower parasite load in bat flies or lower volume and concentration of extracted parasite DNA from ectoparasites. A previous study also found lower prevalence of the *Miniopterus* associated *Polychromophilus melanipherus* in bat flies compared to their hosts [66]. Our results on *Bartonella* suggest that using ectoparasites to detect the presence of vector-borne bacteria in the host population may be more successful than using invasive blood sampling from hosts in this population. In fact, we found *Bartonella* spp. in bat flies in Spain, but not in bats, even though sample size was nearly identical between bats and bat flies (10 and 11). In this instance, positive flies may have fed on infected hosts in the population, prior to switching to an uninfected host that was tested.

Ectoparasite DNA sampling gives access to molecular data for the host, for infectious microorganisms, as well as for the ectoparasite, within one single sample. These samples can be stored long term and used to answer a wide variety of research questions. For instance, Witsenburg et al. (2015) were able to detect the presence of host DNA (*Myotis daubentonii*) in 92.7% of collected bat flies (*Nycteribia kolenatii*) [100]. Hence, not only can the sampling effort be reduced in the field and in the laboratory, but these samples can answer a broad range of interdisciplinary research questions, for example studies of host population genetics [9, 97, 99]. In our study, *Trypanosoma* sequences were identical to a recently reported novel taxa, *Trypanosoma* sp. 1, found in European and African *Miniopterus* spp. [17], hence targeting haematophagous parasites can also reveal diversity and distribution of newly described and undescribed taxa. Furthermore, in some cases the administrative time (such as permit requests for blood sampling) and space for collection storage may also be significantly decreased. Additionally, the appropriate deposition of parasite collections is essential in order to make them more accessible to a broad range of studies [6], which would contribute to reducing unnecessary sampling of hosts, including protected species. Replacing or reducing blood sampling could be especially important in endangered and threatened bat populations. However, it cannot completely replace the need for destructive sampling, such as voucher specimen collection for species description.

Furthermore, this approach can also be broadened to include viral surveillance. In recent years, bat-associated viruses have been successfully detected in bat flies [3, 30, 40, 95]. Since most ectoparasites feed on host blood, samples may also be used for serological detection of antibodies, although sample volume may be challenging. Little is known about the success of identifying antibodies in ectoparasites, but rabies virus antibodies have been successfully detected in blood-sucking reduviid bugs [94].

Another drawback of this method is that data from ectoparasites cannot be attributed to individual bats since bat flies often switch from one host to another [100]. Such data may, however, give access to pathogen distribution among the population of hosts. Monoxenous (parasitising one single host species) bat flies [18], such as the ones we used during this study, are good candidates for this to avoid interspecific host and parasite mixtures. Some bat species are not parasitised by bat flies but other ectoparasitic haematophagous arthropods, such as ticks, mites, fleas or bat bugs can be used to reveal pathogen and parasite diversity in host populations [36–38,68,69,81,103].

Ectoparasites may be used for the detection of non-blood parasites and pathogens as well. For instance, the fungal pathogen *Pseudogymnoascus destructans* [96] has been detected on ectoparasitic *Spinturnix* mites and on nycteribiid bat flies [47, 101]. Additionally, phoretic mites and filarial nematodes have also been observed to infect bat-associated ectoparasites [48, 67]. Hence, ectoparasites may be considered a detection source during surveillance of not strictly blood-associated parasites and pathogens, as well.

In the last few decades, several studies have demonstrated that parasites can be a tool to reveal host population genetic patterns [9, 97, 99] and host migratory and dispersal movements [56, 59, 76, 82]. Moreover, they can be useful in the detection of infectious agent diversity in wide range of wild, captive and domesticated animals [32, 50, 55, 61, 75, 86, 102].

Ectoparasites in historical collections can be useful tools to reveal historical disease patterns and emergence, as well as vector distribution [6, 33]. For instance, the presence of a new haemosporidian parasite species (*Vetufebrus ovatus*) was observed in a streblid bat fly embedded in a Dominican amber [62], which also gives remarkable insights into the evolution and possible vectors of malarial parasites.

Here, we suggest using haematophagous ectoparasites as a tool to reveal the presence and diversity of vector-borne microorganisms, and to replace widely used and invasive methods, such as blood sampling or voucher specimen collection. We emphasise that such samples may be used in a wide variety of studies. Our work emphasises the importance of the study of parasites, which are major contributors to biodiversity [63]. They play an essential role in regulating host populations, for example of invasive species with high competitive strength [64, 77]. They are crucial components of food webs [45, 46]. However, our knowledge is still scarce about their advantageous role in natural systems and it has been shown that they are threatened by climate change and co-extinctions [12, 16, 83]. Recent studies have discussed conservation plan and vulnerability assessment of these species [13, 44]. Our work supports the importance of parasites not only in natural host-parasite systems, but also as a tool in host conservation during pathogen surveillance studies. Therefore, we suggest future conservation efforts should focus not only on hosts but also on the protection of their parasites, particularly in the case of endangered hosts with highly specific parasites. However, we emphasise that voucher specimens and blood sampling may still be important for specific questions. Additionally, we suggest the importance of proper deposition of samples, including vouchers, blood- and parasite samples in museum collections, to make them more accessible and therefore enable a wider range of researchers to gain access to these samples. This would increase the possibility of re-using samples for different studies and therefore reduce the need to resample species. Furthermore, we suggest that future studies should evaluate the use of ectoparasites as a proxy of blood sampling, focusing on different study areas besides pathogen and parasite surveillance.

## Supplementary Material

The Supplementary Material of this article is available at https://www.parasite-journal.org/10.1051/parasite/2020069 *Table S1*. Collection data and infection status of each tested bat and bat fly individual, including date, locality and sex.
*Table S2*. Result of sequence blast searches in NCBI GenBank.

## Conflict of interest

The authors declare that they have no conflict of interest.
