# Supplementary material for: Host conservation through their parasites: molecular surveillance of vector-borne microorganisms in bats using ectoparasitic bat flies
Source: Parasite. 2020 Dec 11;27:72. doi: 10.1051/parasite/2020069 (PMC7731914; doi:10.1051/parasite/2020069)
Supplement: Supplementary file 1 — Table S1. Collection data and infection status of each tested bat and bat fly individual, including date, locality and sex. Table S2. Result of sequence blast searches in NCBI GenBank. [file parasite-27-72-s1.zip › parasite200069-1-olm/200069 Supplementary File 1 EDITOR.pdf]

Supplementary File 1 (PCR protocols):

For ***Bartonella* spp.** detection, a citrate synthase gene *gltA* fragment was targeted (~800 bp), using 443F (5'-GCT ATG TCT GCA TTC TAT CA-3') (Birtles and Raoult, 1996) and BhCS.1137n primers (5'-AAT GCA AAA AGA ACA GTA AAC A-3') (Norman et al., 1995).

Mixture: 25 µl reaction mixture which contained 12.5 µl 2× Green Master Mix (Rovalab GmBH, Teltow, Germany), 6.5 µl water, 1 µl of each primer (0.01 mM final concentration) and 4 µl aliquot of isolated DNA.

Initial denaturation at 94 °C for 5 minutes, followed by 35 cycles of denaturation at 94 °C for 30 seconds, annealing at 48.8 °C for 30 seconds, extension at 72 °C for 1 minute, and a final extension at 72 °C for 5 minutes (443F+BhCS.1137).

For ***Polychromophilus* spp**. detection, a cytochrome b fragment (704 bp) was amplified using the PLAS1 (5'-GAG AAT TAT GGA GTG GAT GGT G-3') and PLAS2 (5'-GTG GTA ATT GAC ATC CWA TCC-3') primers for the first PCR round. For the second round, we used PLAS3 (5'-GGT GTT TYA GAT AYA TGC AYG C-3') and PLAS4 (5'-CATC CWA TCC ATA RTA WAG CAT AG-3') primers (Duval et al., 2007).

Nested PCR Mixtures

First PCR round: 25 µl reaction mixture which contained 0.05 µl Qiagen Taq Polymerase, 0.25 µl dNTP, 0.75 µl of each primer, 5 µl PCR buffer, 1 µl $MgCL_2$, 14.2 µl H_2O and 3 µl aliquot of isolated DNA.

Second PCR round: 25 µl reaction mixture which contained 1 µl of PCR product from first round, 0.05 µl Qiagen Taq Polymerase, 0.25 µl dNTP, 0.75 µl of each primer, 5 µl PCR buffer, 1 µl $MgCL_2$ and 16.2 µl H_2O.

Initial denaturation at 94 °C for 5 minutes, followed by 25 cycles (35 cycles in second round) of denaturation at 94 °C for 30 seconds, annealing at 55°C for 30 seconds, extension at 72 °C for 45 seconds, and a final extension at 72 °C for 10 minutes.

For ***Trypanosoma* spp**., an 18S small-subunit rRNA gene fragment (642 bp) was amplified using the TRYF (5'-CAG AAA CGA AAC ACG GGA G-3') and TRYR (5'-CCT ACT GGG CAG CTT GGA-3') primers for the first PCR round and the SSUF (5'-TGG GAT AAC AAA GGA GCA-3') and SSUR (5'-CTG AGA CTG TAA CCT CAA AGC-3') primers for the second round (Noyes et al., 1999).

Nested PCR Mixtures

First PCR round: 25 µl reaction mixture which contained 0.04 µl Qiagen Taq Polymerase, 0.25 µl dNTP, 0.75 µl of each primer, 5 µl PCR buffer, 0.5 µl $MgCL_2$, 14.71 µl H_2O, and 3 µl aliquot of isolated DNA.

Second PCR round: 25 µl reaction mixture which contained 1 µl of PCR product from first round, 0.04 µl Qiagen Taq Polymerase, 0.25 µl dNTP, 0.75 µl of each primer, 5 µl PCR buffer, 0.5 µl $MgCL_2$ and 16.71 µl H_2O.

Initial denaturation at 94 °C for 5 minutes, followed by 25 cycles (35 cycles in second round) of denaturation at 94 °C for 30 seconds, annealing at 55°C for 30 seconds, extension at 72 °C for 45 seconds, and a final extension at 72 °C for 10 minutes.

PCR products were visualised on 1.5% agarose gel.
